# Female Nurses in Asylum Male Wards

**Published:** 1915-08-21

**Authors:** 


*To the Editor of* The Hospital.

### Female Nurses in Asylum Male Wards.

*To the Editor of* The Hospital.

Sir,—If you will allow me space in your valuable paper, I should like to pass a few comments on Dr. Robertson's letter *re* female nurses in the male wards of asylums. Pray, Sir, excuse any grammatical mistakes I may make, but I feel I must say a few words in defence of the class to which I belong. I must say I very strongly disapprove of the statements made by Dr. Robertson. It seems perfectly clear that he is prejudiced to the male nurses and their efficiency in the wards. The remarks he has thought fit to make are . . . contemptible. . . . When he says that the presence of female nurses in the wards leads very much to the comfort of the patients, I say—and without fear of contradiction—that the male nurses do their duty well, with consideration, tact, and care, and for male mental patients there is no female nurse can possibly understand as a well-trained male nurse can do; and, moreover, no respectable woman ought to be asked to do this class of nursing, and no woman who has any respect for herself would do it for any doctor, no matter who he is.

But the doctor has seized the opportunity when the men are not here to defend themselves. They have done their duty faithfully at home; yes, and they have gone forward to do a greater duty defending you, Sir, and the women, while you try to close the door to their bread and butter when they return, if ever they do. The general tone of your letter suggests that you look forward to installing the female nurses permanently. This might apply to scores of other professions as a result of the war. Are the men to be thrown out because they have done their duty? I fancy you . . . like to be fussed over when you enter the wards. The weaker sex may more readily obey your commands. You may have in view the saving of money, which still furthers the doctor's interests with an ultimate increase in his salary. I am surprised that you have condescended to admit that the male nurses have responded well to their country's call. This is the only good you have to say of them. I am sure the male nurses who have joined the Colours will acquit themselves nobly and well, the same as they do in the wards, with tact and consideration for everyone.

May I say in my opinion there is only one class of male patient the females can nurse well? These are the old, bedridden patients. But I may say that the male nurses can also do this efficiently and well, and, if I may say so, intelligently. Then, again, the female nurses cannot take part in the various games and pursuits of the male patients. This is not fair to the patient, and must retard his recovery to a certain extent. A man understands a man; he will trust his attendant and confide things to him he would never do to a female nurse. All this leads to his comfort and well-being, and to his ultimate recovery. I do not wish to complain of the nurses, they are doing their duty well; in the meantime they have no wish to do this work. But they will continue to do what they can while our men return from the battlefields with honour and glory. Dr. Robertson seems to forget all about the good works the male attendants have done in years gone by.—Yours respectfully,

A Trained Male Nurse.

[Certain adjectives and sentences have been necessarily omitted. They do not strengthen the argument, being mere personalities. The questions at issue have a far higher bearing and importance than our correspondent apprehends.—Ed. The Hospital.]

